# Vaccination of metastatic melanoma patients with autologous dendritic cell (DC) derived-exosomes: results of thefirst phase I clinical trial

**DOI:** 10.1186/1479-5876-3-10

**Published:** 2005-03-02

**Authors:** Bernard Escudier, Thierry Dorval, Nathalie Chaput, Fabrice André, Marie-Pierre Caby, Sophie Novault, Caroline Flament, Christophe Leboulaire, Christophe Borg, Sebastian Amigorena, Catherine Boccaccio, Christian Bonnerot, Olivier Dhellin, Mojgan Movassagh, Sophie Piperno, Caroline Robert, Vincent Serra, Nancy Valente, Jean-Bernard Le Pecq, Alain Spatz, Olivier Lantz, Thomas Tursz, Eric Angevin, Laurence Zitvogel

**Affiliations:** 1 Department of Immunotherapy, Institut Gustave Roussy, Villejuif, France; 2 Department of Clinical Oncology, Institut Curie, Paris, France; 3 ERM0208 INSERM, Department of Clinical Biology, Institut Gustave Roussy, Villejuif, France; 4 U520INSERM, Institut Curie, Paris, France; 5 Cell Therapy Unit, Institut Gustave Roussy, Villejuif, France; 6 Anosys S.A, Evry, France; 7 Anosys Inc, Menlo Park, California, USA; 8 Department of Pathology, Institut Gustave Roussy, Villejuif, France; 9 Department of Dermatology, Institut Gustave Roussy, Villejuif, France

**Keywords:** exosomes, dendritic cells, phase I trial, cancer vaccine, immunotherapy

## Abstract

**Background:**

DC derived-exosomes are nanomeric vesicles harboring functional MHC/peptide complexes capable of promoting T cell immune responses and tumor rejection. Here we report the feasability and safety of the first Phase I clinical trial using autologous exosomes pulsed with MAGE 3 peptides for the immunization of stage III/IV melanoma patients. Secondary endpoints were the monitoring of T cell responses and the clinical outcome.

**Patients and methods:**

Exosomes were purified from day 7 autologous monocyte derived-DC cultures. Fifteen patients fullfilling the inclusion criteria (stage IIIB and IV, HLA-A1^+^, or -B35^+ ^and HLA-DPO4^+ ^leukocyte phenotype, tumor expressing MAGE3 antigen) were enrolled from 2000 to 2002 and received four exosome vaccinations. Two dose levels of either MHC class II molecules (0.13 versus 0.40 × 10^14 ^molecules) or peptides (10 versus 100 μg/ml) were tested. Evaluations were performed before and 2 weeks after immunization. A continuation treatment was performed in 4 cases of non progression.

**Results:**

The GMP process allowed to harvest about 5 × 10^14 ^exosomal MHC class II molecules allowing inclusion of all 15 patients. There was no grade II toxicity and the maximal tolerated dose was not achieved. One patient exhibited a partial response according to the RECIST criteria. This HLA-B35^+^/A2^+ ^patient vaccinated with A1/B35 defined CTL epitopes developed halo of depigmentation around naevi, a MART1-specific HLA-A2 restricted T cell response in the tumor bed associated with progressive loss of HLA-A2 and HLA-BC molecules on tumor cells during therapy with exosomes. In addition, one minor, two stable and one mixed responses were observed in skin and lymph node sites. MAGE3 specific CD4^+ ^and CD8^+ ^T cell responses could not be detected in peripheral blood.

**Conclusion:**

The first exosome Phase I trial highlighted the feasibility of large scale exosome production and the safety of exosome administration.

## Background

Immunization protocols rely on the capacity of the vaccine design to elicit long term protective, peptide specific, MHC restricted-CD4^+ ^and CD8^+ ^T cell responses in cancer patients. In preclinical studies, few vaccination strategies were shown to counteract tumor induced-tolerance and to promote immunity to cancer leading to tumor eradication [1]. One of the most promising approach recently developped is based on the adoptive transfer of mature dendritic cells (DC) pulsed with tumor peptides and control antigen [2,3]. Dhodapkar et al. performed a pionnering clinical trial demonstrating the rapid generation of T cell immunity against recall antigens and Keyhole Limpet Hemocyanin in normal volunteers [4] and Schüler-Thurner et al. successfully promoted active, melanoma peptide-specific, IFNγ producing, effector CD8^+ ^T cells [5,6] and helper CD4^+ ^Th1 lymphocytes [6] in the majority of patients with metastatic melanoma. If a single injection of CD34+ progenitor derived-DC vaccine can lead to induction of T cell immunity [7], correlations between clinical responses and CTL Tc1/CD4^+ ^Th1 activation have been rarely reported [8-10]. Therefore, it is conceivable that antigen spreading initiated by the specific T cells and/or intervention of alternate effectors also elicited by mature DC [11,12] might account for this apparent discrepancy.

DC process exogenous antigens in endosomal compartments such as multivesicular endosomes [13] which can fuse with plasma membrane, thereby releasing antigen presenting vesicles called «exosomes» [14-16]. Exosomes are 50–90 nm diameter vesicles containing antigen presenting molecules (MHC class I, class II, CD1, hsp70–90) tetraspan molecules (CD9, CD63, CD81), adhesion molecules (CD11b, CD54) and CD86 costimulatory molecules [17-19] i.e the necessary machinery required for generating potent immune responses. Exosomal MHC class I and II /peptide complexes are functional but require to be transferred to naive DC [20-23] to promote T cell activation leading to tumor eradication [16,21]. Exosomes pulsed with tumor peptides are more efficient than peptides alone and as efficient as mature DC for the priming of MART1 -specific CTL and for tumor growth inhibition in the HLA-A2 transgenic mouse model [21]. The molecular characterization of DC derived-exosomes [24,25] and the definition of quality control parameters for exosome purification and storage [26] allowed to conduct a Phase I clinical trial aimed at evaluating the feasability of exosome harvesting from autologous monocyte derived-DC cultures and the safety of exosome inoculation in melanoma patients. Secondary endpoints were the immunomonitoring of peptide specific CD4^+ ^and CD8^+ ^T cell responses restricted by exosomal MHC class II and I molecules respectively. Up to 41 ± 6.7 (9–115) id/sc vaccinations/patient at the lowest dosage (i.e 0.13 × 10^14 ^exosomal MHC class II molecules /vaccine) could be generated for all patients from one leukapheresis. One partial response and some other tumor regressions at skin and lymph node sites even in tumors that did not respond to other vaccine formulations were observed in the absence of toxicity. A case report of MART1 antigen spreading and MHC class I loss variant suggested that exosomes mediated bioactivity in vivo, supporting to conduct Phase II clinical trials.

## Patients, Material and Methods

### Protocol design, Patients' characteristics and eligibility criteria

**Figure 1:**
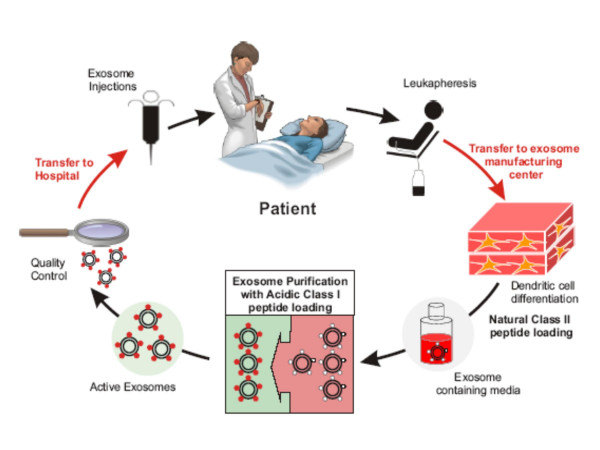
**Study design**. Different weeks (W) of the study are described. Screening, HLA typing and tumor evaluation were performed within 2 weeks before lymphapheresis. DTH (Multi Mérieux skin test) was performed at the time of lymphapheresis, and exosome vaccine started 3 weeks later.

**Figure 2:**
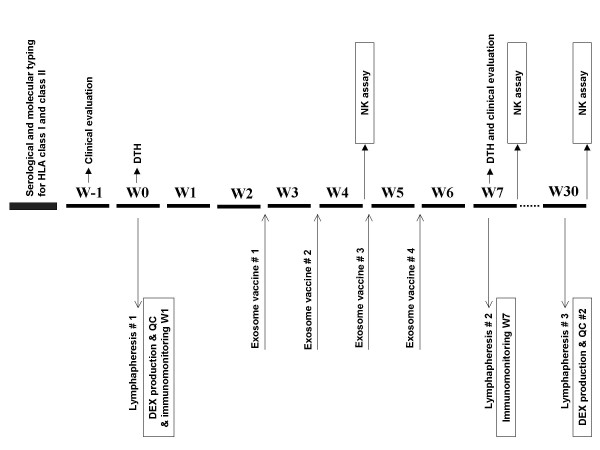
**Schema of exosome purification processes**. Exosomes were purified from monocyte derived-DC (MD-DC) culture supernatants according to a good manufacturing process already described [26]. In the second part of the trial, indirect loading of MHC class II peptides (MAGE3_247–258_.DP04, KKLLTQHFVQENYLEY) was performed on DC cultures followed, after exosome purification by a direct loading of MHC class I (MAGE3_168–176_.A1/B35; EVDPIGHLY) peptides at 10 or 100 μg/ml at pH 4.2 [22]. Quality control parameters included dosing of exosomal MHC class II molecules, flow cytometry analyses and functional assays using superantigens as described in material and methods.

We report here about the first phase I trial (Fig. 1) which administers 4 exosome vaccinations intradermally and subcutaneously at 1 week intervals (Fig. 2). The study was approved by the Ethics Committee, local IRBs and regulatory authorities, and informed written consent was given by all patients. The authors were in compliance with the Helsinki Declaration. Fifteen patients bearing melanoma fullfilling the inclusion criteria were enrolled in the study (see Additional file 1 and Table 1). Inclusion criteria were: biopsy-proven American Joint Committee on Cancer stage IIIB and IV metastatic melanoma, HLA-A1^+^, or -B35^+ ^and HLA-DPO4^+ ^phenotype (as defined by serological and molecular typing), tumor expressing MAGE3 antigen (assessed by RT-PCR, as described elsewhere [27,28]), inclusion at least 4 weeks after cytotoxic chemotherapy, surgery, or radiation therapy, intradermal skin test positivity to one or more recall antigens using Pasteur Mérieux multi DTH test, age > 18 years, Karnofsky performans status >80%, lymphocyte counts >1000 /mm^3^. Exclusion criteria were: prior chemotherapy or biotherapies <4 weeks before trial entry, brain metastasis, pregnancy, concurrent steroids or immunosuppressive therapy, history of asthma, congestive heart failure, autoimmune disease, active infections. Three patients presenting with skin or LN involved sites declined conventional therapies and received exosomes upfront (see Additional file 1 and Table 1). Patients received a 4 week outpatient vaccination course with antigen loaded DC derived-exosomes given intradermally (1/10^th^) and subcutaneously (9/10^th^) every week for a total of 4 vaccinations (Fig. 2). The injection sites were rotating between both thighs and forearms. The study was scheduled in two steps i.e 1) MHC class I peptide loading was «indirectly» performed on the DC culture at 10 μg/ml, and 2) MHC class I peptide loading was «directly» performed onto purified and acid eluted exosomes (Fig. 1). Indeed, our preclinical studies aimed at comparing the exosome immunogenicity after indirect versus direct peptide loading showed a superiority of the latter process [20,22]. Exosomes (quantified as concentrations of MHC class II molecules, see below) or peptides that were pulsed onto exosomes were administered in a dose escalation design. In the first step, 0.13 or 0.40 × 10^14 ^MHC class II molecules were inoculated in a cohort of three patients each. In the second step, the concentration of peptide loading onto exosomes varied from 10 μg/ml to 100 μg/ml in a cohort of 3 and 6 patients respectively (see Additional file 1 and Table 1).

**Table 1:** Formulation of Product

Dose Groups	Peptides loaded / HLA class	Peptide loading method and concentration	DEX dose (expressed as numbers of MHC class II molecules)
A	MAGE A3 (168–176) / class I	Indirect (10 μg/mL)	0.13 × 10^14^
	MAGE A3 (247–258) / class II	Indirect (10 μg/mL)	
	tetanus toxoid / class II	Indirect (10 μg/mL)	
B	MAGE A3 (168–176) / class I	Indirect (10 μg/mL)	0.4 × 10^14^
	MAGE A3 (247–258) / class II	Indirect (10 μg/mL)	
C	MAGE A3 (168–176) / class I	Direct (10 μg/mL)	0.13 × 10^14^
	MAGE A3 (247–258) / class II	Indirect (10 μg/mL)	
	tetanus toxoid / class II	Indirect (10 μg/mL)	
D	MAGE A3 (168–176) / class I	Direct (100 μg/mL)	0.13 × 10^14^
	MAGE A3 (247–258) / class II	Indirect (10 μg/mL)	

### Clinical grade exosome production

#### MD-DC propagation

Based on size and density, we could rapidly purify exosomes from monocyte derived-DC (MD-DC) culture supernatants according to a good manufacturing process already described [26]. Briefly, the adherent fraction of peripheral blood mononuclear cells (PBMC) from a leukapheresis is replenished with fresh medium (AIMV media discarded from haptoglobin and albumin through a 500 kDa MWCO hollow fiber cartridge ultrafiltration (UFP-500-C-4A from A/G Technology, Needham, MA) followed by sterile filtration through a 0.22 um Sartopore 2 membrane) containing rhuGM-CSF (50 ng/ml, Immunex, Seattle, WA) and rhu IL-4 (250 IU/ml, Schering Plough, Kennilsworth, NJ) until day 7.

#### Clinical grade exosome purification

Exosomes secreted in the supernatant of MD-DC cultures (1–4 liters) were purified to 175 fold the starting volume according to a GMP method previously described [26].

#### MHC class I peptide loading of exosomes

This method has been previously described [21,22]. Briefly, the indirect loading of peptides was performed for both MHC class I (Mage3_168–176_.A1/B35; EVDPIGHLY) and MHC class II peptides (Mage3_247–258_.DP04, KKLLTQHFVQENYLEY) in pharmaceutical quality (Multiple Peptide Systems, San Diego, CA) at 10 μg/ml at day 6 of the MD-DC culture and the exosomes were purified from day 7 culture supernatant as described above. The direct loading of MHC class I peptides at pH 4.2 has been reported [22].

### Quality control parameters for exosome inoculation

Insurance quality criteria allowing exosome release in patients were qualitative (expression of CD81 tetraspanins), quantitative (at least 1 × 10^14 ^MHC class II molecules in immunocapture assays) and included a functional assay (bioactivity in the superantigen SEE test of potency).

#### Quantitation of exosome MHC class II concentration by adsorption ELISA and immunophenotyping

These methods have been previously described [26].

#### Test of potency

The bioactivity of exosomal MHC class II molecules was tested using a superantigen bioassay. Exosomes were incubated with femtoM dosages of SEE and washed by density cushion. SEE harboring exosomes were pulsed onto Raji cells. Exosome pulsed Raji cells were subjected to Jurkat cell lines that produced IL-2 in response to SEE. IL-2 concentrations in the supernatants of the Raji/Jurkat cocultures were assessed using a commercial IL-2 ELISA kit.

### Clinical monitoring

Adverse events were graded according to the WHO Toxicity criteria. All patients underwent assessment of tumor status at baseline and 2 weeks after the fourth exosome vaccination. Tumor evaluation was performed using the RECIST criteria. Disease progression was defined as >20% increase in target lesions and/or the appearance of new lesions, partial response as a > 30% decrease in the sum of the longest diameters of target lesions.

### Immunomonitoring assays

Pre-, per- and post-immunization PBMCs were collected before vaccination (week 0, 5% of total leukocytes collected from the starting leukapheresis), after 2 (week 4, 50 ml heparinized blood sample) and 4 vaccines (week 7, by leukapheresis), isolated by Ficoll density gradient and conditioned either for immediat testing or kept frozen for further analyses. In patient #12, single cell suspensions from dissociated cervical lymph nodes resected at the end of the induction and retreatment phases were also collected.

#### Immunophenotyping

Determination of peripheral lymphocytes subsets were performed by four color immunostainings using combinations of FITC-, PE-, Cychrome-, APC-labeled mAbs directed against CD2, CD3, CD4, CD5, CD8, CD19, CD25, CD27, CD45RA, CD56, CD69, CD122, HLA-DR (all purchased from Becton Dickinson, Pont de Claix, FR). Controls included isotype-matched immunoglobulins. Thawed PBMCs were stained for 15 min at 4°C, washed twice and fixed in 1X PBS with 0.1% paraformaldhehyde. Ten thousand viable PBMCs were acquired on a FACSCalibur cytometer (Becton Dickinson, BDIS, San Jose, CA, USA) according to standard FSC/SSC criteria and analyzed with the Cellquest software.

#### Peptides and recall antigens for functionnal assays

Reactivity of PBMCs was assessed in response to MAGE3_168–176_.A1/B35, MAGE3_247–258_.DP04 (Multiple Peptide System), and control viral peptides (HIV/nef_113–128_.A1, HIV/nef_73–82_.B35, FLU/pb1_591–599_.A1, FLU/np_265–274_.A2, EBV/bzlf1_54–64_.B35, EBV/bmlf1_259–267_.A2, CMV/pp65_495–503_.A2 TT/p2_830–844_, Eurogentec, Seraing, BEL) used according to the HLA classe I phenotype of patients, as well as, against tetanus anatoxin (at 100, 10 and 1 μg/ml), tuberculin (at 50, 5 and 0.5 IU/ml), and phytohemaglutinin (at 2.5, 0.5, and 0.1 μg/ml, PHA HA16, Murex Biotech, Dartford, UK).

#### Proliferation assays

Fresh PBMCs (2 × 10^5 ^cells/well) were cultured in triplicates in presence of graded doses of peptides (at 50, 5, and 0.5 μg/ml) or control antigens or medium alone. Proliferative capacity was determined after overnight pulsing with [^3^H]thymidine (1μCi/well, NEN, Paris, France) at day 5 of the coculture (earlier at day 3 for PHA). Cells were harvested onto 96-well Unifilter microplates, dried overnight and radioactivity counted on a microplate scintillation counter (TopCount-NXT, Packard, CA, USA). All determinations were made in triplicate wells and data were calculated as means ± SEM of cpm.

#### Enzyme-linked Immunospot assays

ELISPOT assay for the detection of antigen-specific IFNγ-producing T cells was performed as described previously [29]. Briefly, fresh or frozen PBMCs (5 × 10^5^cells/well) were cultured in triplicate in nitrocellulose-bottomed 96-well plates (MAHA S4510, Millipore, Saint Quentin- en-Yvelines, FR) precoated with 2 μg/ml of a primary anti-IFNγ mAb (1-D1K, Mabtech, Hamburg, GER) in presence of peptides (at 5 μg/ml) or control antigens in RPMI1640 medium supplemented with 8% human AB serum. After incubation for 48 hrs, wells were washed five times and incubated with a secondary biotinylated anti-IFNγ mAb (7-B6-1, Mabtech) for 2 h, washed and stained using an extravidine-alkaline phosphatase conjugate substrate kit (Biorad, Hercules, CA, USA). Spots were evaluated and counted using a computer-assisted video imaging analyser (Bioreader 2000, Biosys, Karben, GER).

#### Microcultures for semiquantitative detection of Mage3.A1/B35 CTL precursors

The microculture method developed by Coulie et al. [30] was used to assessed CTL precursors specific for MAGE3.A1/B35 peptide. Briefly, groups of 2 × 10^5 ^peptide-pulsed PBMC (20 μg/ml) were cultured in Iscove's medium with 10% human AB serum supplemented with IL-2 (20 IU/ml), IL-4 (10 ng/ml) and IL-7 (10 ng/ml) and restimulated at day 7 with replacement of 50% fresh medium with addition of MAGE3.A1/B35 peptide (10 μg/ml). On day 15, microcultures were screened for specific CD8^+ ^T cells using the relevant MAGE3.A1 or B3501 tetramers coupled to PE together with a control APC labeled-HLA-A1 containing an influenza peptide (all tetramers kindly given by D. Colau, Ludwig Institut for Cancer Research, Brussels, BE). This microculture procedure was also applied on patient #12's tumor infiltrating LN to highlight CD8+ T cells specific for HLA-A2/MART1_26–35_, and HLA-B3501/MAGE3_168–176_. Flow cytometry acquisition and analysis of tetramer positive cells were performed as recommanded by P. Coulie [30].

### Immunohistochemistry on lymph node tissues

Immunostainings were performed on sections obtained from formalin-fixed and paraffin-embedded lymph node samples. Sections were deparaffinized, placed in 10 mmol/L Na-citrate buffer (pH.7), and heated in a microwave during 20 min. Endogenous peroxidase was blocked with 1% hydrogen peroxide in methanol for 30 min. Slides were stained using anti-CD3 and anti-CD57 mAb (Pharmingen, France) and secondary antibodies with appropriate controls.

## Results

### Feasability of exosome production in advanced melanoma patients

Leukaphereses of 1.5 blood mass performed in the 15 metastatic melanoma patients enrolled in the study allowed the recovery of 9.7 × 10^9 ^± 0.8 PBMC (range: 4.4–15) containing 20.7% ± 1.8 CD14^+ ^cells (range: 12.4–33.0). These monocytes differentiated into immature MD-DC in rhu GM-CSF and rhu IL-4 (means: 313 × 10^6 ^± 100, range: 50–1200) as assessed at day 7 in flow cytometry highlighting loss of CD14 molecules, acquisition of CD1a, poor cell surface expression of CD83. In 3 patients out of 15, the first leukapheresis did not allow CD14^+ ^cells to adhere and consequently, a second leukapheresis was required to harvest exosomes. The GMP process allowed to purify a mean of about 5.22 × 10^14 ^± 0.9 (range: 1.2–15.0) exosomal MHC class II molecules from supernatants of day 6 to day 7 MD-DC required for up to 41 ± 6.7 (9–115) id/sc vaccinations/patient at the lowest dosage (i.e 0.13 × 10^14 ^exosomal MHC class II molecules /vaccine). As for the patients who benefited from continuation treatment requiring a second leukapheresis (pt #3, #12 and #14), the second exosome recovery was not significantly different from the first one. The quality control parameters required for GMP exosome batch release are reminded in M&M.

GMP exosomes were successfully produced from DC cultures derived from all melanoma patients enrolled in this phase I study, allowing at least 4 exosome inoculations.

### Safety and clinical outcome

No major (>grade II) toxicity was observed. A grade 1 fever was recorded in 5 patients. We noted slight inflammatory reactions at vaccine sites without outward delayed type hypersensitivity reactions. Some patients (#4, #8, #11, #12 and #15) reported a transient swelling and sensitivity of cutaneous and lymph nodes metastases 48 hrs after each exosome inoculation.

**Figure 3:**
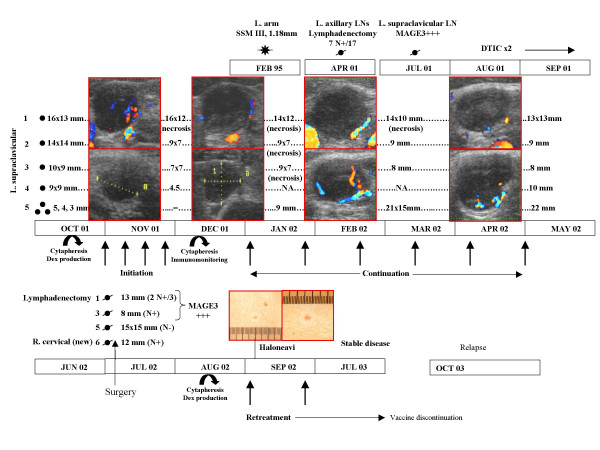
**Clinical outcome of patient #12 during exosome-based vaccination**. This patient presented with progressive supraclavicular lymph nodes containing MAGE3 expressing tumor cells in July 2001 when enrolled in the exosomes Phase I trial starting in October 2001. She underwent a first leukapheresis for exosomes production and vaccination (weekly injections in Nov. 2001 during induction therapy and from January 2002 to April 2002 on a three week basis in continuation treatment). The initial size of the target LN are indicated on the left and were followed up by doppler pulsed ultrasonography from Nov 2001 to Dec 2001 (sizes indicated on the right side at the end of the induction therapy). Continuation therapy with exosomes was indicated and maintained clinical stability until the last available exosome dose in Jul. 2002, when she underwent surgery for lymphadenectomy. Results obtained by the pathologists are indicated (N+ if node is invaded by tumor cells, N- if not, MAGE3+ as expression of MAGE3 mRNA in RT-PCR). A second leukapheresis was performed on Aug. 2002 allowing a second therapy with exosomes on a 3 week basis that was continuated until Jul 2004. Six months after exosomes discontinuation (Oct 2004), the patient relapsed in contralateral LN and presented with one lung metastasis.

Among the six patients vaccinated in the first part of the study, only one patient (#3) presenting with a stage III disease exhibited a minor response (disappearance of one sc lesion out of 3). This patient benefited from a continuation therapy with exosomes every other 3 weeks for 21 months and remained stable for up to24 months. It is noteworthy that this patient was progressing despite vaccination with MAGE3 protein prior to enrollment in the Phase I exosome trial. In the second part of the study («direct loading»), responses were only observed in the second group of patients receiving the highest dosages of peptides (100 μg/ml) pulsed onto exosomes. One female patient (#12) presenting with five supraclavicular invaded lymph nodes after conventional chemotherapy (DTIC, 2 cycles) exhibited a partial response (PR) after the induction therapy. Interestingly, disappearance of arterial neovasculature concomittant with tumor shrinkage and necrosis as assessed by doppler pulsed ultrasonography could be demonstrated (Fig. 3). Halo of depigmentation around neavi appeared 10 months after the initiation of vaccination (Fig. 3). A continuation therapy with exosomes was administered for 4 months every other 3 weeks allowing stabilisation without toxicity supporting the indication of surgery that confirmed the partial response (Jul. 2002, Fig. 3) followed by a second leukapheresis allowing to pursue vaccination for 10 months. This patient relapsed in contralateral nodes six months after exosomes discontinuation but exhibited a slow pace of tumor growth. Two additionnal patients (#11, #14) presenting with only skin and /or LN lesions exhibited transient stabilisation and started a continuation therapy. Interestingly, patient # 14 who displayed a SD with exosomes had previously progressed despite biotherapy with IFNα and vaccination with poxviruses recombinant for the MAGE 3A1 cDNA. It is of note that patient #9 in a M1b stage exhibited a regression on a subcutaneous nodule but did progress in the pulmonary sites (mixed response).

Exosome therapy promoted 2 stable diseases, 1 minor response, 1 partial response and 1 mixed response in skin or LN sites even in patients progressing with biotherapies or alternate vaccines. Patient #3 and #12 with respectively minor and partial responses are still alive. Neither of these patients exhibited a delayed type hypersensitivity response to the immunizing epitopes after the completion of the 4 vaccines.

### T cell immunomonitoring

**Figure 4:**
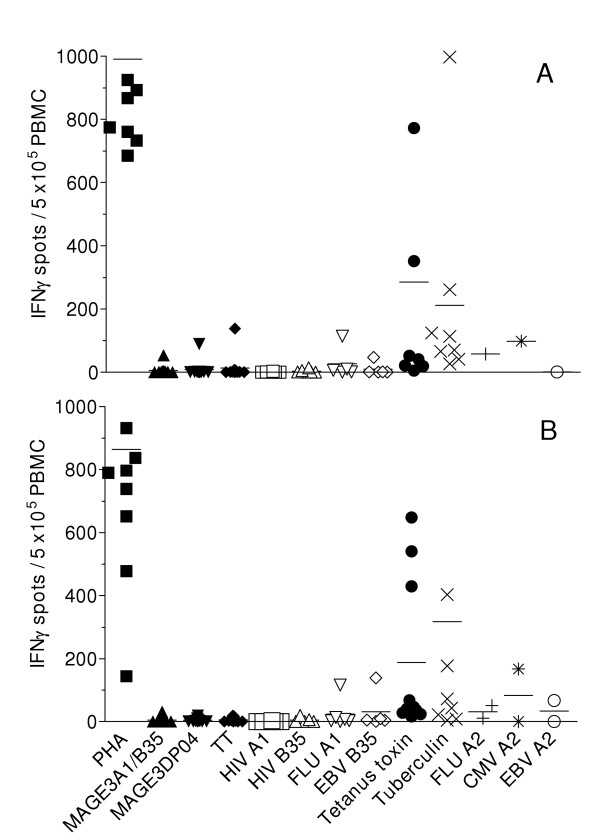
**Evaluation of Tc1/Th1 immune responses to melanoma and viral/recall antigens**. A. Before exosome vaccination (W1). B. After exosome inoculation (W7). PBMC obtained at baseline (W1) and after 4 exosome injections are cultured 48 h with the immunizing melanoma antigens i.e Mage 3.A1/B35 or Mage3.DP04 (5 μg/ml) or with viral/recall control antigens (FluMP.A1, HIV.A1, EBV.B35, tetanus anatoxin, tuberculin) or with PHA. The three first patients were also assayed with the universal MHC class II restricted TT peptide. The specific T cell response in each of the evaluated patients is expressed as the number of IFNγ spot forming unit/5 × 10^5 ^PBMC.

The phenotypic analyses of lymphocyte subsets did not reveal any significant changes in the percentages nor absolute counts following exosome therapy (not shown). Interestingly, the CD122 molecule i.e IL-2Rβ chain was upregulated in both CD4 and CD8 T cell subsets after exosome therapy (p < 0.05 using Student t'test, week 7 versus week 1 for CD4+ T cells). Serial ex vivo ELISPOT analyses of PBMC before (W1, Fig. 4A) and after (W7, Fig. 4B) exosome vaccination did not reveal any significant Th1 (using TT or DP04 MAGE 3 peptides) or Tc1 (using MAGE 3. A1/B35 peptides) type immune responses induced by exosomes. Recall responses (proliferative responses to TT protein using autologous DC or restimulation with autologous DC pulsed with MAGE 3.DP04) did not allow to conclude that exosomes boosted MHC class II specific responses at these time points (not shown). In accordance with these findings, the skin reactivities to iterative injections of id/sc exosomes did not correspond to clinically significant Delayed Type Hypersensitivity responses. Extensive immunomonitoring by screening of 15 day-microcultures of MAGE3 peptide pulsed PBMC performed in the presence of IL-2, IL-4, IL-7 [30] using the MAGE3.A1/B35 tetramers allowed to detect and clone specific CTL precursors only in 3 patients (#3, 9 and 11) but estimated at a low frequency (10^-6 ^- 10^-7 ^of the CD8^+ ^T cells). These studies did not allow to conclude for significant anti-MAGE3A1/B35 CTL responses induced by exosome therapy nor to suggest the clonal expansion of discrete T cell specificities (not shown) in patients' peripheral blood.

### Comprehensive analyses of a partial response

**Figure 5:**
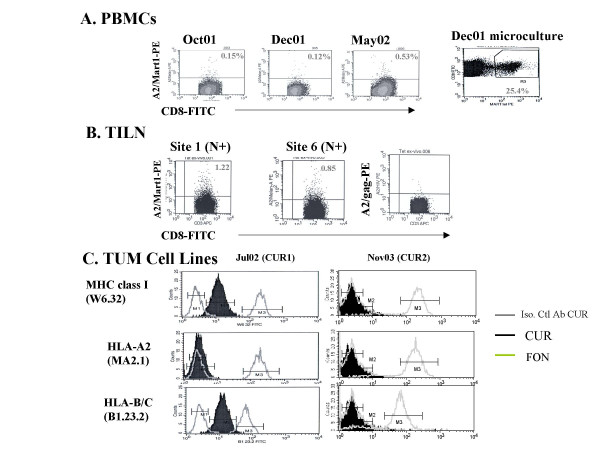
**Antigen spreading and MHC class I loss variant in patient#12**. Flow cytometry analyses on serial blood specimen (A) or tumor invaded lymph nodes (B) gating on CD8+ T lymphocytes stained using A2/Mart1 or A2/gag specific fluorescent tetramers. Ex vivo microcultures stimulated with Mart1 peptides and examined according to similar settings. (C) Flow cytometry analyses of two CUR tumor cell lines (pt#12) after the first exosome therapy (continuation treatment) in clinical response and after the second exosome course (second leukapheresis) at relapse for MHC class I (anti-HLA-A2 mAb, anti-HLA-BC mAb and W6.32 Ab) expression. A positive control was included which consisted of a allogeneic HLA-A*0201melanoma line FON.

Patient#12 presenting with five supraclavicular lymph nodes exhibited a partial regression after 4 exosome vaccines in November 2001 and underwent a continuation therapy from Jan. 2002 to April 2002 (Fig. 3). The HLA class I haplotype of the patient was A2.A26.B35.B44 and the initial tumor sites expressed both MAGE1, MAGE3 and other melanoma antigens such as Melan-A/MART1 and NA-17 (as assessed using semiquantitative RT-PCR). The antitumor effects did not correlate with an enhanced frequency of MAGE3 specific CTL precursors as demonstrated using microculture detection assays after the first 4 vaccines or after the continuation treatment (not shown). However, tumor shrinkage after the continuation therapy prompted a partial surgical resection in July 2002 and comprehensive analyses of tumor infiltrating lymphocytes (TILN) and tumor cells in 4 avalaible sites (#1, 3, 5, 6 in Fig. 3). Site 5 was scored tumor free by the pathologists. Site 1 and 3 maintained the transcription of Mage3 mRNA. In these TILN sites, detection of HLA-A2/MART1_26–35_, -/gp100_209M-217 _-/Tyrosinase_239–251 _and HLA-B3501/MAGE3_168–176 _-specific CD8+ lymphocytes using soluble fluorescent tetramers were performed directly *ex_vivo *and after microculture restimulation assays using MART1_26–35 _or MAGE3 peptides.Only high frequencies of MART1 specific CD8+ T cells were detected in TILN from site 1 and 6 (1.22 and 0.85 % respectively) and in peripheral blood (0.53% in May 2002 versus 0.15% in Oct. 2001) that were confirmed by expansion in all microculture assays (Fig. 5A and 5B). In parallel, halo of depigmentation around naevi appeared (Fig. 3). Moreover, a cell line established from site 1 (CUR1) did not express the HLA-A2 allele in flow cytometry using the MA2.1mAb but maintained the expression of HLA-BC molecules (Fig. 5C). In November 2003 while disease was slowly progressive, another LN was removed allowing to re-establish another cell line (CUR2) that no longer express HLA-BC molecules (as assessed in flow cytometry using W6.32 or anti-HLA-BC mAb, Fig. 5C).

**Figure 6:**
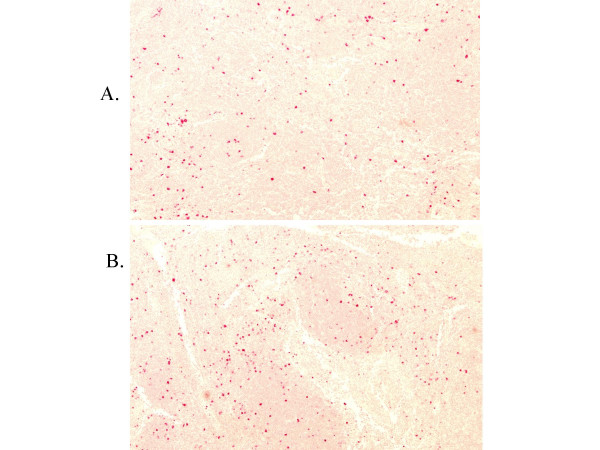
**Lymphocyte recruitment and activation in patient#12's melanoma**. T cells that were double-stained by anti-CD3 and anti- CD57 mAb were counted in 2 sections of 6 lymph nodes available from the lymph node dissection specimen obtained before (A. April 2001) and after (B. July 2002) treatment (cf Fig. 3). Counts were performed in 12 sections in total as follows: cells that were double-stained by CD3 and CD57 were counted on the whole section including B cell areas, and the total count was reported to 1 mm^2^. Results showed an increase of the CD3+CD57+ cells after treatement (mean = 122/mm^2^) as compared to the count before treatment (mean: 58/mm^2^).

Immunohistochemistry performed on the primary (April 2001) and secondary (July 2002) tumor specimen revealed quantitative modifications of tumor infiltrating CD3+CD57+ T cells. While activated T cells were mostly surrounding the tumor bed initially, a two fold expansion or recruitment of activated T cells invade tumor areas after exosome vaccines (Fig. 6).

## Discussion

This work is reporting for the first time the feasability and safety of DC derived-exosome-based vaccination in melanoma patients. From a single leukapheresis, up to 10^14^-10^15 ^exosomal MHC class II molecules could be purified in GMP conditions for all patients, allowing 9–123 sc/id vaccinations with 0.13 × 10^14 ^exosomal MHC class II molecules. The optimal dosages of exosomal MHC class II molecules required to trigger an efficient peptide-specific CTL response leading to tumor rejection was about 10^10^-10^11 ^molecules in preclinical studies in the HLA-A2 transgenic mouse model [21]. The optimal method for pulsing exosomal MHC class I molecules with peptides was a direct loading after acid elution of the exosomal pellet [20,22]. A dose response was observed in vitro, prompting the use of 100 ug/ml for peptide pulsing onto GMP exosomes. As to MHC class II associated-peptide loading, pulsing immature day 5 DC with 10 ug/ml was optimal [22]. However, in this Phase I study, since no untoward clinical effect nor pharmacodynamic parameter appear to be dose-dependent, neither the peptide pulsing method nor the dosage of exosomes or peptides could help defining the maximal tolerated doses of exosomes. Interestingly, in the second Phase I trial initiated at the Dukes' University in unresectable lung carcinoma, there was a trend for a better efficacy of exosomes directly pulsed with MHC class I peptides in long term survival [31].

Only one objective response (PR) according to the RECIST criteria was recorded. However, one minor response and two stabilisations prompted the continuation of treatment as well. Tumor regression was exclusively observed in skin and lymph node lesions, as already reported for peptide-based vaccines [32]. It is noteworthy that three of these patients had primarily resisted to alternate immunotherapy strategies (i.e IFN type I, ALVAC-Mage 3, Mage 3 protein) but it is also likely that such immunomodulators might have facilitated exosome efficacy. However, we were not able to detect significant Mage 3 specific CTL precursors in peripheral blood (nor lymph nodes and lesions) after exosome vaccination. Notewithstanding, MHC class I tumor loss variant and naevi depigmentation was observed in one case (pt #12), suggesting melanoma antigen spreading mediated by HLA-A2-restricted CTL cells. It is conceivable that the Mart1-specific CTL monitored in blood and in LN TIL could have mediated part of these antitumor effects.

No DTH responses to iterative inoculations of exosomes or MHC class II restricted-direct or recall responses (to TT epitopes (3 pts tested), or Mage DP04 epitopes (12 patients tested)) were detected at 4 and 6 weeks of exosome vaccination. It is noteworthy that peptides and not whole proteins (such as TT or KLH) were used to pulse DC in vitro, in contrast to what has been reported in most DC trials. However, in the transgenic Marilyn mouse model [23], id inoculation of I-A^b ^harboring exosomes pulsed with H-Y epitopes could promote expansion of peptide specific CD4^+ ^transgenic T cells. Nevertheless, MHC class I restricted-CTL responses elicited by exosomes pulsed with Mart1 peptides in HLA-A2 transgenic mice were dramatically boosted by adjuvants [21]. Therefore, it is conceivable that injection of exosomes in the area of an inflammatory draining lymph node harboring mature APC might promote elicitation of T cell immune responses leading to tumor regressions in patients. Alternatively, we recently showed that regulatory C4+CD25+ T cells (Treg) restrict T cell responses elicited by DC derived-exosomes (Chaput et al, in preparation). Therefore, high yields of Treg at start might severely impair the efficacy of exosomes to prime or boost T cell responses. Supporting this view, our unpublished data demonstrate synergistic T cell dependent-antitumor effects between peptide pulsed-exosomes and immunopotentiating dosing of cyclophosphamide.

Since no specific CD4+ or CD8+ T cells generated by the exosome vaccines could be detected and could account for the tumor regression in patient#12, what could have been the primary effectors accounting for antigen spreading? We were able to show enhanced NK cell effector functions following exosome administration in peripheral blood of 8/13 patients including patient #12 for whom CD3-CD57+ cells expressing PEN5 (mostly expressed on CD56^dim ^NK cells) were markedly infiltrating the tumor after therapy (N. Chaput, manuscript in preparation). Therefore, it is conceivable that DC derived-exosomes from melanoma patients are specifically endowed with NK cell stimulatory capacity in vivo. This hypothesis deserves further investigations and should be pursued in the next clinical studies. Moreover, since our preclinical data in tumor bearing mice highlighted a critical role of suppressor T cells in restricting exosome-mediated specific T cell responses, T regulatory cells should be monitored and combination of cyclophosphamide together with exosomes could be invisioned (N. Chaput, in preparation). Phase II/III trials will address the potential of exosomes to enhance time to progression in advanced non small cell lung carcinoma patients.

## Abbreviations

**DC**: Dendritic cells; **MD-DC**: monocyte-derived DC; **Dex: **Dendritic cell -derived exosomes; **CTL: **Cytotoxic T Lymphocytes; **GMP: **good manufacturing processes; **MHC: **Major Histocompatibility Complex; **LN**: lymph nodes.

## Authors' contributions

Bernard Escudier^1• ^was the principal investigator,

Thierry Dorval^2•^, Sophie Piperno^2^, Caroline Robert^3,9 ^were the clinicians ensuring patients' enrolment, follow up and clinical care.

^• ^BE and TD equally contributed to this work

Marie-Pierre Cabi^4^, Sophie Novault^3^, Christophe Leboulaire^3^, Mojgan Movassagh^3 ^and Christophe Leboulaire^3 ^were monitoring T cell responses corrdinated by Olivier Lantz^4 ^and Eric Angevin^1^

Nathalie Chaput^3^, Fabrice André^3 ^and Caroline Flament^3^monitored NK cell responses

Catherine Boccaccio was in charge of the cell therapy unit at the Institut Gustave Roussy^5^

Sebastian Amigorena^4^, Christian Bonnerot^4 ^and Laurence Zitvogel^1 ^were the scientists at the source of preclinical data orienting the study design.

Thomas Tursz^1 ^was heading the Host and Tumor Development Programm of the Institut Gustave Roussy, initiating the clinical study.

Vincent Serra^6^, Nancy Valente^7^, Olivier Dhellin^6 ^were the Director of Anosys SA, the medical director of Anosys Inc. and the Pharmacist in charge of production, regulatories and quality insurance in Anosys SA respectively. Jean-Bernard Le Pecq^6,7^was the Chief and Scientific Officer of Anosys leading the research and development of the exosome programm.

Alain Spatz^8^, Christophe Borg3 were in charge of the immunohistochemitry analyses on tumor specimen.

## Supplementary Material

Additional File 1 Dosing, demographic, base line data and clinical outcome Click here for file
