# Supplementary material for: Vaccination of metastatic melanoma patients with autologous dendritic cell (DC) derived-exosomes: results of thefirst phase I clinical trial
Source: J Transl Med. 2005 Mar 2;3:10. doi: 10.1186/1479-5876-3-10 (PMC554765; doi:10.1186/1479-5876-3-10)
Supplement: Additional File 1 — Dosing, demographic, base line data and clinical outcome [file 1479-5876-3-10-S1.doc]

| **Dosing, Demographic and Baseline data** | | | | | | | | | | **Clinical Outcome** | | | | | |
| --- | --- | --- | --- | --- | --- | --- | --- | --- | --- | --- | --- | --- | --- | --- | --- |
| Patient # | Formulation ^a^ | Number of Injections | HLA | Prior treatment | TNM/Stage | Age/Sex | Metastatic Sites | | Status at entry | TTP from  Leukapheresis (days) | TTP from  #1 Injection (days) | Survival from  Leukapheresis (days) | Survival from  #1 Dex injection (days) | Clinical Response | |
|  |  |  |  |  |  |  | M1a | M1b |  |  |  |  |  | After 4 injections | Duration of Responses |
| 02-001 | A | 4 | A1 | Ch,S,R | T2N1M3/IV | 30/M | LNs | Liver /Bone | PD | 57 | 29 | 92 | 64 | PD |  |
| 02-002 | A | 4 | A1 | Ch,Im,S,O | TNM0/IIIc | 36/M | Skin |  | PD | 69 | 35 | 206 | 172 | PD |  |
| 03-003**^c^** | A & D | 22 | B35 | S, Im | T0N2M0/IIIb | 57/F | Skin |  | PD | 477 | 449 | 1034**+ ^d^** | 1006**+ ^d^** | SD **^f g^** | 24 months |
| 02-004 | B | 4 | B35 | S | TN1M3/IV | 58/M | LNs | Lung | PD | 61 | 36 | 194 | 169 | PD |  |
| 02-005 | B | 4 | B35 | Ch,S,Im | T4N3M1/IV | 63/M | Skin |  | PD | 62 | 37 | 446 | 421 | PD |  |
| 02-006 | B | 4 | A1/B35 | Ch,S,Im | T1N0M1/IV | 49/M | LNs |  | PD | 69 | 43 | 188 | 162 | PD |  |
| 02-007 | C | 4 | A1 | S | T4N2CM0/IIIb | 66/F | Skin |  | PD | 68 | 43 | 491 | 466 | PD |  |
| 02-008 | C | 4 | B35 | Ch,S | T3N0M3/IV | 60/F |  | Liver/Soft tissue | PD | 69 | 44 | 298 | 273 | PD |  |
| 03-009 | C | 4 | A1 | Ch,S,Im | T4N2M1b/IV | 63/M | Skin/LNs | Lung/Pleura | PD | 53 | 29 | 66**+^e^** | 42**+^e^** | PD **^h^** |  |
| 02-010 | D | 4 | A1 | Ch,S | T1M0M3/IV | 61/F | Skin | Breast/liver | PD | 58 | 33 | 181 | 156 | PD |  |
| 02-011 | D | 6 | A1 | S | T2N2CM0/IIIc | 41/F | Skin |  | PD | 85 | 57 | 247 | 219 | SD **^f^** | 2 months |
| 02-012 | D | 25 | B35 | Ch,S | T2N3M2/IV | 49/F | LNs |  | PD | 154 | 128 | 824**+^d^** | 798**+ ^d^** | PR **^f^** | 16 months |
| 02-013 | D | 4 | A1 | Ch,S | T3BN1M3/IV | 62/F | Skin | Lung/Liver/Pancreas | SD | 50 | 28 | 427 | 405 | PD |  |
| 03-014 | D | 7 | A1 | Ch,S,Im | T0N2M0/IIIb | 45/F | Skin/LNs |  | PD | 148 | 120 | 468**+^d^** | 440**+^d^** | SD **^f^** | 2 months |
| 02-015 | D | 4 | A1 | S,Im | T1N2M1A/IV | 40/M | Skin |  | PD | 62 | 36 | 334 | 308 | PD |  |

**^a^** Formulation of Product (see Table 1)

**^b^** Prior treatment: Ch=chemotherapy, S=surgery, R=radiotherapy, Im=immunotherapy, O=other

**^c^** Patient 03-003 received 12 treatments of formulation A and 10 treatments of formulation D

**+**Four patients were censored at their last date of known survival status.

**^d^** patients still alive

**^e^** one was lost to follow up

**^f^** These patients received a continuation treatment

**^g^**minor response

**^h^**mixed response
